# Supplementary material for: Similarity-based transfer learning with deep learning networks for accurate CRISPR-Cas9 off-target prediction
Source: PLoS Comput Biol. 2025 Oct 24;21(10):e1013606. doi: 10.1371/journal.pcbi.1013606 (PMC12571277; doi:10.1371/journal.pcbi.1013606)
Supplement: S1 Text — (PDF) [file pcbi.1013606.s001.pdf]

# S1 Text: Detailed description of the considered ML and DL models and their hyperparameters

## 1 Scikit-Learn models

We hereby present briefly the four Scikit-Learn models we developed: a One-Layer Perceptron, a Two-Layer Perceptron, a Random Forest classifier, and a Logistic Regression classifier. The Scikit-Learn models are used as benchmark in our experiments to evaluate the performance gain (if any) between machine learning models and deep learning models.

**One Hidden Layer Perceptron (MLP1):** A one hidden layer perceptron is a three-layer perceptron. It is a type of feedforward artificial neural network with three distinct layers: an input layer, a hidden layer, and an output layer. The input layer receives the 7×L matrices as input data, the hidden layer processes intermediate representations, and the output layer produces the off-target predictions. The activation function applied to the neurons in the hidden and output layers is a Rectified Linear Unit (ReLU) activation function [1].

**Two Hidden Layer Perceptron (MLP2):** A two hidden layer perceptron extends the architecture of a three-layer perceptron by introducing an additional hidden layer. It is thus often referred as a four-layer perceptron. The four-layer perceptron allows for more complex representations of data. Each hidden layer processes intermediate features, enabling the network to capture intricate patterns and non-linear relationships. The activation function applied to the neurons in the hidden and output layers is a Rectified Linear Unit (ReLU) activation function [1].

**Random Forest (RF) classifier:** An RF classifier is an ensemble learning method that combines multiple decision trees to improve predictive accuracy and reduce overfitting. Each decision tree in the forest is trained on a random subset of the data, and the final prediction is obtained by aggregating the individual tree predictions. [2].

**Logistic Regression (LR) Classifier:** An LR classifier models the probability of an instance belonging to a particular class. The logistic function (sigmoid) maps linear combinations of input features to probabilities within the range of values [0, 1]. In this study, we used the standard L2 regularization technique [3].

## 2 Deep Neural Networks with TensorFlow

Here, we describe three types of deep neural networks used in our study: (i) FNNs (Feedforward Neural Networks), (ii) CNNs (Convolutional Neural Networks), and (iii) RNNs (Recurrent Neural Networks). As standard ML models, these networks can be employed for transfer learning in the context of off-target predictions in genome editing.

**Feed-forward Neural Networks (FNNs)** We implemented 3 different FNN models for our experiments: a three-layer FNN (FNN3), a five-layer FNN (FNN5), and a ten-layer FNN (FNN10). We used 7×L matrices containing sgRNA-DNA information as input. The networks begin with a first dense layer that collects information from the sgRNA-DNA matrices. Before hyperparameter tuning, the Feedforward Neural Networks (FNNs) are constructed such that the dense layers are distinct from the dropout and batch normalization layers. Specifically, the three-layer FNN consists of three dense layers, each accompanied by a dropout layer and a batch normalization layer. Similarly, the five-layer and ten-layer FNNs consist of five dense layers and ten dense layers, respectively, followed by a dropout layer and a batch normalization layer. By default, all dense layers use a uniform kernel initializer and a Rectified Linear Unit (ReLU) activation function, except for the final layer, which employs a softmax activation.

**Convolutional Neural Networks (CNNs)** We implemented three different Convolutional Neural Network (CNN) models: a three-layer CNN (CNN3), a five-layer CNN (CNN5), and a ten-layer CNN (CNN10). Similar to FNNs, we used as input 7×L matrices containing sgRNA-DNA information. All networks begin with a 2-dimensional convolutional layer. The differences between CNN3, CNN5, and CNN10 emerge at the second layer. Specifically:

- CNN3: After the initial convolutional layer, CNN3 includes a 2-dimensional max pooling layer, followed by a batch normalization layer, and a dropout layer. Next, a flatten layer is employed, leading to a dense layer, another batch normalization layer, and a dropout layer. Finally, the network reaches the last layer, consisting of a single neuron.

- CNN5: In addition to the initial convolutional layer, CNN5 introduces another 2-dimensional convolutional layer as the second layer. Following this, a max pooling layer, batch normalization, and dropout layer are applied. The architecture then features two blocks of dense layers, each accompanied by batch normalization and dropout layers. The final dense layer contains a single neuron.

- CNN10: Up to the flatten layer, CNN10 shares the same architecture as CNN5. However, beyond the flatten layer, CNN10 incorporates seven blocks of dense layers, each accompanied by batch normalization and dropout layers.

By default, all dense layers employ a uniform kernel initializer and a Rectified Linear Unit (ReLU) activation function.

**Recurrent Neural Networks (RNNs)** We implemented two different Recurrent Neural Network (RNN) models: a three-layer Long Short-Term Memory (LSTM) model and a three-layer Gated Recurrent Unit (GRU) model. For the LSTM model, the initial block consists of an LSTM layer, followed by a batch normalization layer and a dropout layer. Similarly, for the GRU model, the first block includes a GRU layer, followed by batch normalization and dropout layers. Beyond the first block, both the LSTM and GRU models share an identical architecture. A dense layer is employed, followed once again by a batch normalization layer and a dropout layer,

before reaching the final layer comprising a single neuron. All layers use by default a ReLU activation function.

# References

[1] Rudolf Kruse, Sanaz Mostaghim, Christian Borgelt, Christian Braune, and Matthias Steinbrecher. Multi-layer perceptrons. In *Computational intelligence: a methodological introduction*, pages 53–124. Springer, 2022.

[2] Leo Breiman. Random forests. *Machine learning*, 45:5–32, 2001.

[3] Jerome Friedman, Trevor Hastie, and Robert Tibshirani. *The elements of statistical learning*, volume 1. Springer series in statistics New York, NY, USA:, 2001.
