# Supplementary material for: Similarity-based transfer learning with deep learning networks for accurate CRISPR-Cas9 off-target prediction
Source: PLoS Comput Biol. 2025 Oct 24;21(10):e1013606. doi: 10.1371/journal.pcbi.1013606 (PMC12571277; doi:10.1371/journal.pcbi.1013606)
Supplement: S1 Table — (PDF) [file pcbi.1013606.s002.pdf]

# S1 Table: Tables reporting hyperparameters of the considered ML and DL models

Table A: Hyperparameters for machine learning models for CD33 dataset. If a parameter is not mentioned specifically, we used the parameter by default of the model implementation in the *scikit_learn* library.

| Model | Parameters |
|---|---|
| MLP1 | learning_rate: invscaling, hidden_layer_sizes: 227, early_stopping: False, activation: tanh |
| MLP2 | learning_rate: invscaling, hidden_layer_sizes: (216, 25)), early_stopping: False, activation: tanh |
| RF | n_estimators: 483, criterion: entropy |
| LR | solver: newton-cg, penalty: none |

Table B: Hyperparameters for machine learning models for CIRCLE dataset. If a parameter is not mentioned specifically, we used the parameter by default of the model implementation in the scikit_learn library.

| Model | Parameters |
|---|---|
| MLP1 | learning_rate: constant, hidden_layer_sizes: 72, early_stopping: False, activation: logistic |
| MLP2 | learning_rate: constant, hidden_layer_sizes: (216, 75)), early_stopping: False, activation: logistic |
| RF | n_estimators: 183, criterion: entropy |
| LR | solver: sag, penalty: none |

Table C: Hyperparameters for machine learning models for SITE dataset. If a parameter is not mentioned specifically, we used the parameter by default of the model implementation in the scikit_learn library.

| Model | Parameters |
|---|---|
| MLP1 | learning_rate: invscaling, hidden_layer_sizes: 94, early_stopping: False, activation: logistic |
| MLP2 | learning_rate: constant, hidden_layer_sizes: (250, 25)), early_stopping: False, activation: logistic |
| RF | n_estimators: 784, criterion: entropy |
| LR | solver: sag, penalty: l2, C : 0.233572 |

Table D: Hyperparameters for deep neural networks for CD33 dataset. If a parameter is not mentioned specifically, we used the parameter by default of the model implementation in the TensorFlow library.

| Model | Parameters |
|---|---|
| FFN3 | unit_layer_1: 64, unit_layer_2: 200, unit_layer_3: 5, unit_dropout_1: 0.3, is_batch_normalization_1: True, unit_batch: 64 |
| FFN5 | unit_layer_1: 200, unit_layer_2: 75, unit_layer_3: 256, unit_layer_4: 8, unit_layer_5: 128, unit_dropout_1: 0.1, unit_dropout_2: 0.1, is_batch_normalization_1: True, is_batch_normalization_2: True, unit_batch: 64 |
| FFN10 | unit_layer_1: 200, unit_layer_2: 100, unit_layer_3: 32, unit_layer_4: 256, unit_layer_5: 32, unit_layer_6: 75, unit_layer_7: 128, unit_layer_8: 256, unit_layer_9: 200, unit_layer_10: 128, unit_dropout_1: 0.1, unit_dropout_2: 0.2, unit_dropout_3: 0.1, unit_dropout_4: 0.05, unit_batch: 64, is_batch_normalization_1: True, is_batch_normalization_2: True, is_batch_normalization_3: True, is_batch_normalization_4: True, is_batch_normalization_5: True, is_batch_normalization_6: True, |
| CNN3 | unit_layer_1: 100, unit_layer_2: 256, activation_layer_1: relu, activation_layer_2: relu, activation_layer_3: sigmoid, unit_dropout_1: 0.1, unit_dropout_2: 0.1, is_batch_normalization_1: True, is_batch_normalization_2: True, unit_batch: 256 |
| CNN5 | unit_layer_1: 100, unit_layer_2: 200, unit_layer_3: 64, unit_layer_4: 75, activation_layer_1: relu, activation_layer_2: tanh, activation_layer_3: relu, activation_layer_4: relu, activation_layer_5: sigmoid, unit_dropout_1: 0.1, unit_dropout_2: 0, unit_batch: 32, is_batch_normalization_1: True, is_batch_normalization_2: True, is_batch_normalization_3: True, |
| CNN10 | unit_layer_1: 256, unit_layer_2: 128, unit_layer_3: 100, unit_layer_4: 256, unit_layer_5: 64, unit_layer_6: 32, unit_layer_7: 8, unit_layer_8: 64, unit_layer_9: 75, activation_layer_1: relu, activation_layer_2: relu, activation_layer_3: tanh, activation_layer_4: tanh, activation_layer_5: relu, activation_layer_6: relu, activation_layer_7: relu, activation_layer_8: tanh, activation_layer_9: tanh, activation_layer_10: sigmoid, unit_dropout_1: 0.15, unit_dropout_2: 0.1, unit_dropout_3: 0.05, unit_dropout_4: 0.15, unit_dropout_5: 0.15, unit_dropout_6: 0.05, is_batch_normalization_1: True, is_batch_normalization_2: True, is_batch_normalization_3: True, is_batch_normalization_4: True, is_batch_normalization_5: True, is_batch_normalization_6: True, is_batch_normalization_7: True, unit_batch: 64 |
| LSTM | unit_layer_1: 200, unit_layer_2: 256, activation_layer_1: relu, activation_layer_2: relu, activation_layer_3: sigmoid, unit_dropout_1: 0.15, unit_dropout_2: 0, unit_batch: 256, is_batch_normalization_1: True, is_batch_normalization_2: True, |
| GRU | unit_layer_1: 256, unit_layer_2: 64, activation_layer_1: tanh, activation_layer_2: tanh, activation_layer_3: sigmoid, unit_dropout_1: 0.1, unit_dropout_2: 0.1, unit_batch: 32, is_batch_normalization_1: True, is_batch_normalization_2: True, |

Table E: Hyperparameters for deep neural networks for CIRCLE dataset. If a parameter is not mentioned specifically, we used the parameter by default of the model implementation in the TensorFlow library.

| Model | Parameters |
|---|---|
| FFN3 | unit_layer_1: 200, unit_layer_2: 8, unit_layer_3: 2, unit_dropout_1: 0.3, is_batch_normalization_1: True, unit_batch: 32 |
| FFN5 | unit_layer_1: 128, unit_layer_2: 128, unit_layer_3: 32, unit_layer_4: 75, unit_layer_5: 200, unit_dropout_1: 0.3, unit_dropout_2: 0.15, is_batch_normalization_1: True, is_batch_normalization_2: True, unit_batch: 128 |
| FFN10 | unit_layer_1: 200, unit_layer_2: 200, unit_layer_3: 8, unit_layer_4: 64, unit_layer_5: 200, unit_layer_6: 75, unit_layer_7: 200, unit_layer_8: 32, unit_layer_9: 32, unit_layer_10: 200, unit_dropout_1: 0.3, unit_dropout_2: 0.2, unit_dropout_3: 0.1, unit_dropout_4: 0.3, is_batch_normalization_1: True, is_batch_normalization_2: True, is_batch_normalization_3: True, is_batch_normalization_4: True, is_batch_normalization_5: True, is_batch_normalization_6: True, unit_batch: 256 |
| CNN3 | unit_layer_1: 200, unit_layer_2: 75, activation_layer_1: relu, activation_layer_2: tanh, activation_layer_3: sigmoid, unit_dropout_1: 0.15, unit_dropout_2: 0.1, is_batch_normalization_1: True, is_batch_normalization_2: True, unit_batch: 64 |
| CNN5 | unit_layer_1: 256, unit_layer_2: 256, unit_layer_3: 32, unit_layer_4: 75, activation_layer_1: relu, activation_layer_2: tanh, activation_layer_3: relu, activation_layer_4: tanh, activation_layer_5: sigmoid, unit_dropout_1: 0.1, unit_dropout_2: 0.15, unit_batch: 32, is_batch_normalization_1: True, is_batch_normalization_2: True, is_batch_normalization_3: True, |
| CNN10 | unit_layer_1: 128, unit_layer_2: 256, unit_layer_3: 128, unit_layer_4: 128, unit_layer_5: 32, unit_layer_6: 75, unit_layer_7: 75, unit_layer_8: 64, unit_layer_9: 32, activation_layer_1: relu, activation_layer_2: tanh, activation_layer_3: tanh, activation_layer_4: relu, activation_layer_5: relu, activation_layer_6: tanh, activation_layer_7: tanh, activation_layer_8: relu, activation_layer_9: relu, activation_layer_10: sigmoid, unit_dropout_1: 0.05, unit_dropout_2: 0.1, unit_dropout_3: 0, unit_dropout_4: 0, unit_dropout_5: 0.1, unit_dropout_6: 0.05, is_batch_normalization_1: True, is_batch_normalization_2: True, is_batch_normalization_3: True, is_batch_normalization_4: True, is_batch_normalization_5: True, is_batch_normalization_6: True, is_batch_normalization_7: True unit_batch: 512 |
| LSTM | unit_layer_1: 64, unit_layer_2: 100, activation_layer_1: tanh, activation_layer_2: tanh, activation_layer_3: sigmoid, unit_dropout_1: 0.1, unit_dropout_2: 0.25, unit_batch: 32 is_batch_normalization_1: True, is_batch_normalization_2: True, |
| GRU | unit_layer_1: 32, unit_layer_2: 128, activation_layer_1: tanh, activation_layer_2: tanh, activation_layer_3: sigmoid, unit_dropout_1: 0.1, unit_dropout_2: 0.05, is_batch_normalization_1: True, is_batch_normalization_2: True, unit_batch: 64 |

Table F: Hyperparameters for deep neural networks for SITE dataset. If a parameter is not mentioned specifically, we used the parameter by default of the model implementation in the TensorFlow library.

| Model | Parameters |
|---|---|
| FFN3 | unit_layer_1: 128, unit_layer_2: 75, unit_layer_3: 16, unit_dropout_1: 0.3, is_batch_normalization_1: True, unit_batch: 512 |
| FFN5 | unit_layer_1: 8, unit_layer_2: 32, unit_layer_3: 128, unit_layer_4: 200, unit_layer_5: 32, unit_dropout_1: 0.05, unit_dropout_2: 0.2, is_batch_normalization_1: True, is_batch_normalization_2: True, unit_batch: 512 |
| FFN10 | unit_layer_1: 256, unit_layer_2: 100, unit_layer_3: 200, unit_layer_4: 8, unit_layer_5: 100, unit_layer_6: 75, unit_layer_7: 128, unit_layer_8: 75, unit_layer_9: 100, unit_layer_10: 128, unit_dropout_1: 0.2, unit_dropout_2: 0.05, unit_dropout_3: 0.1, unit_dropout_4: 0.2, unit_batch: 128, is_batch_normalization_1: True, is_batch_normalization_2: True, is_batch_normalization_3: True, is_batch_normalization_4: True, is_batch_normalization_5: True, is_batch_normalization_6: True, |
| CNN3 | unit_layer_1: 100, unit_layer_2: 64, activation_layer_1: tanh, activation_layer_2: relu, activation_layer_3: sigmoid, unit_dropout_1: 0.15, unit_dropout_2: 0, unit_batch: 64, is_batch_normalization_1: True, is_batch_normalization_2: True, |
| CNN5 | unit_layer_1: 100, unit_layer_2: 100, unit_layer_3: 32, unit_layer_4: 16, activation_layer_1: relu, activation_layer_2: tanh, activation_layer_3: relu, activation_layer_4: tanh, activation_layer_5: sigmoid, unit_dropout_1: 0.15, unit_dropout_2: 0.05, is_batch_normalization_1: True, is_batch_normalization_2: True, is_batch_normalization_3: True, unit_batch: 32 |
| CNN10 | unit_layer_1: 200, unit_layer_2: 128, unit_layer_3: 128, unit_layer_4: 100, unit_layer_5: 75, unit_layer_6: 8, unit_layer_7: 64, unit_layer_8: 64, unit_layer_9: 8, activation_layer_1: relu, activation_layer_2: relu, activation_layer_3: tanh, activation_layer_4: tanh, activation_layer_5: relu, activation_layer_6: tanh, activation_layer_7: relu, activation_layer_8: tanh, activation_layer_9: tanh, activation_layer_10: sigmoid, unit_dropout_1: 0.1, unit_dropout_2: 0.05, unit_dropout_3: 0, unit_dropout_4: 0.05, unit_dropout_5: 0, unit_dropout_6: 0.15, is_batch_normalization_1: True, is_batch_normalization_2: True, is_batch_normalization_3: True, is_batch_normalization_4: True, is_batch_normalization_5: True, is_batch_normalization_6: True, is_batch_normalization_7: True unit_batch: 128 |
| LSTM | unit_layer_1: 256, unit_layer_2: 256, activation_layer_1: relu, activation_layer_2: relu, activation_layer_3: sigmoid, unit_dropout_1: 0.1, unit_dropout_2: 0.25, unit_batch: 64, is_batch_normalization_1: True, is_batch_normalization_2: True, |
| GRU | unit_layer_1: 32, unit_layer_2: 200, activation_layer_1: tanh, activation_layer_2: relu, activation_layer_3: sigmoid, unit_dropout_1: 0.05, unit_dropout_2: 0.15, unit_batch: 128, is_batch_normalization_1: True, is_batch_normalization_2: True, |
