# Supplementary material for: Similarity-based transfer learning with deep learning networks for accurate CRISPR-Cas9 off-target prediction
Source: PLoS Comput Biol. 2025 Oct 24;21(10):e1013606. doi: 10.1371/journal.pcbi.1013606 (PMC12571277; doi:10.1371/journal.pcbi.1013606)
Supplement: S1 Fig — (PDF) [file pcbi.1013606.s003.pdf]

S1 Fig: Precision-Recall curves for three source datasets

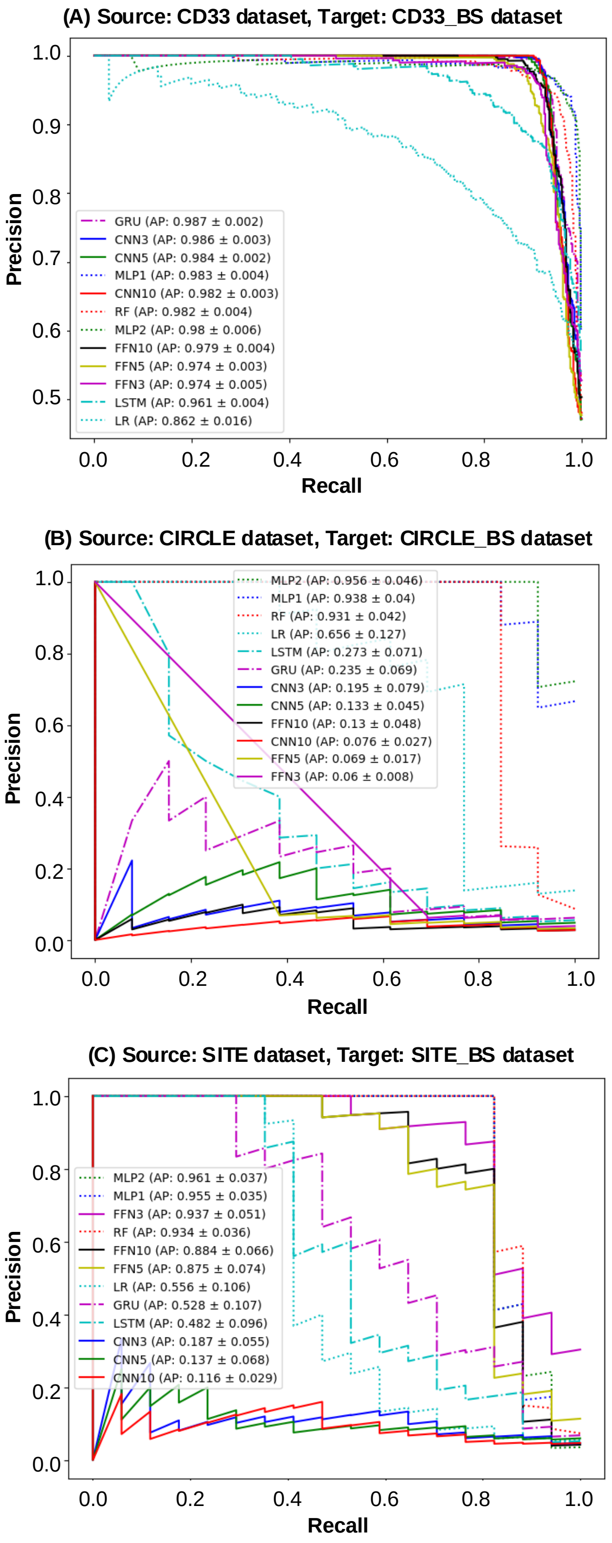

Figure A: Precision-Recall curves for model evaluation. Precision-Recall curves for models trained on: (A) CD33 dataset, (B) CIRCLE dataset, and (C) SITE dataset used as source and evaluated on their bootstrapped target counterparts.

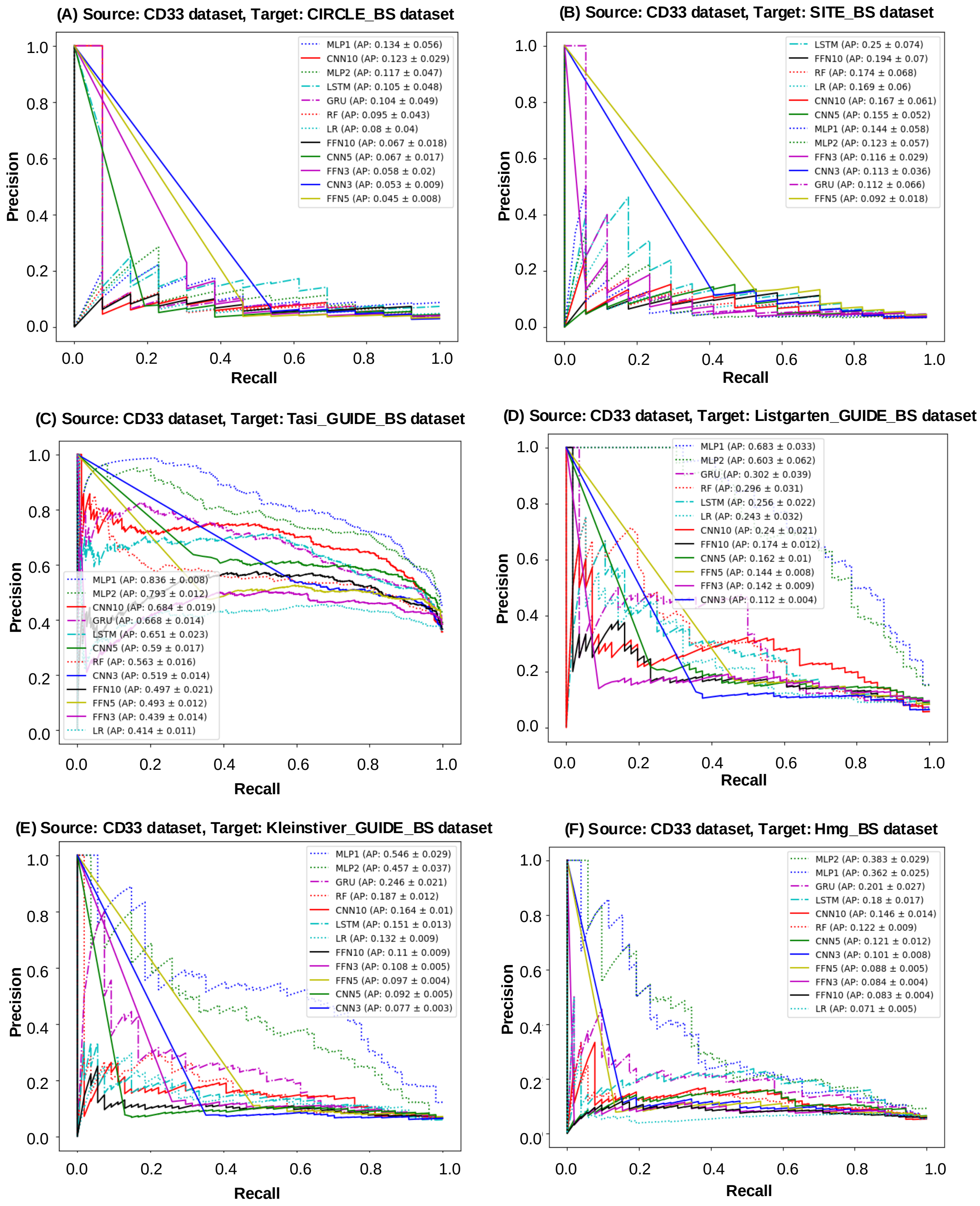

Figure B: Precision-Recall curves for model evaluation - CD33 dataset. Precision-Recall curves for the CD33 dataset, used as source, and six bootstrapped datasets: CIRCLE_BS, SITE_BS, Tasi_GUIDE_BS, Listgarten_GUIDE_BS , Kleinstiver_GUIDE_BS, Hmg_BS, used as target. The Precision values for each model are displayed in descending order within each figure.

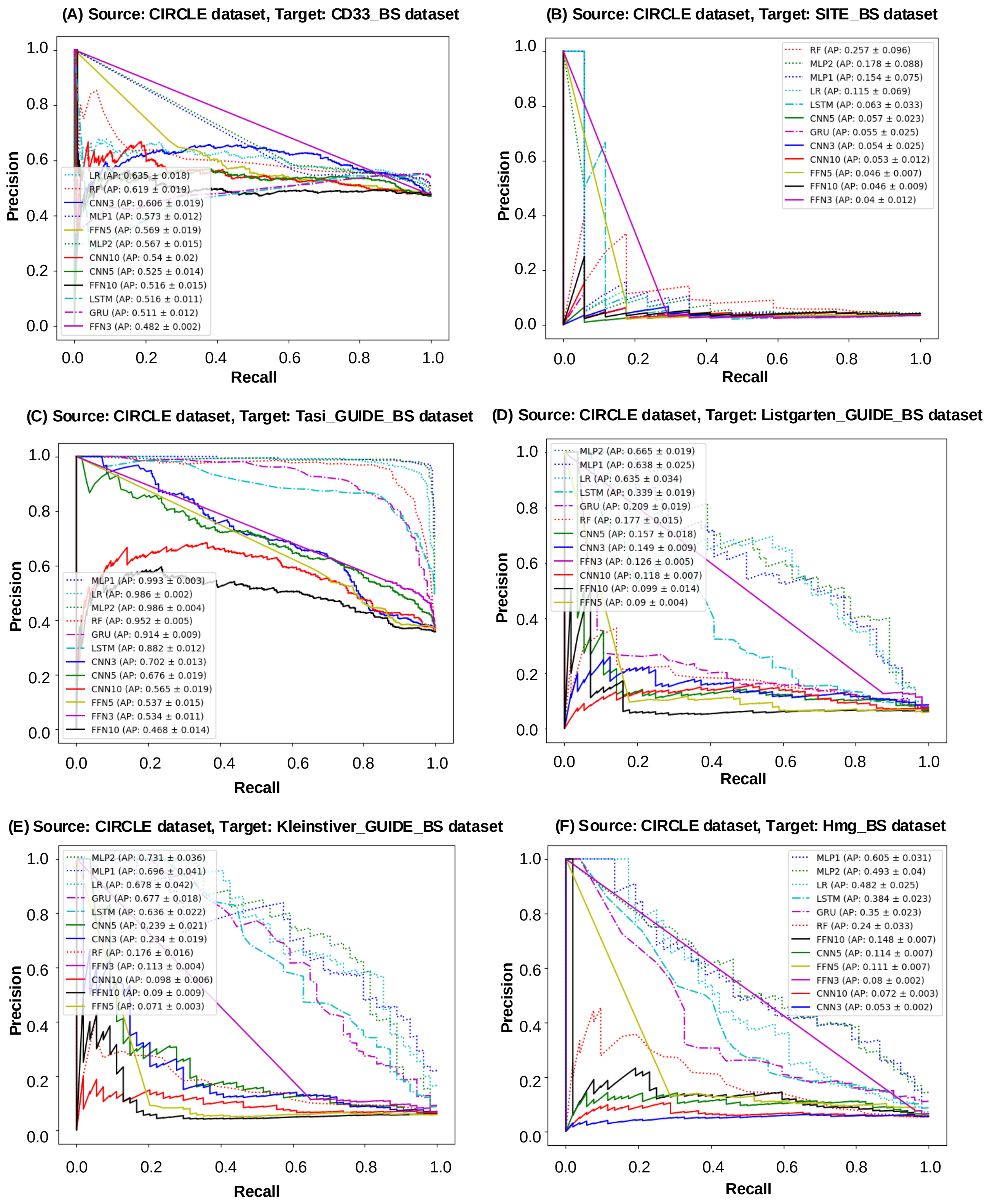

Figure C: Precision-Recall curves for model evaluation - CIRCLE dataset. Precision-Recall curves for the CIRCLE dataset, used as source, and six bootstrapped datasets: CD33_BS, SITE_BS, Tasi_GUIDE_BS, Listgarten_GUIDE_BS , Kleinstiver_GUIDE_BS, Hmg_BS, used as target. The Precision values for each model are displayed in descending order within each figure.

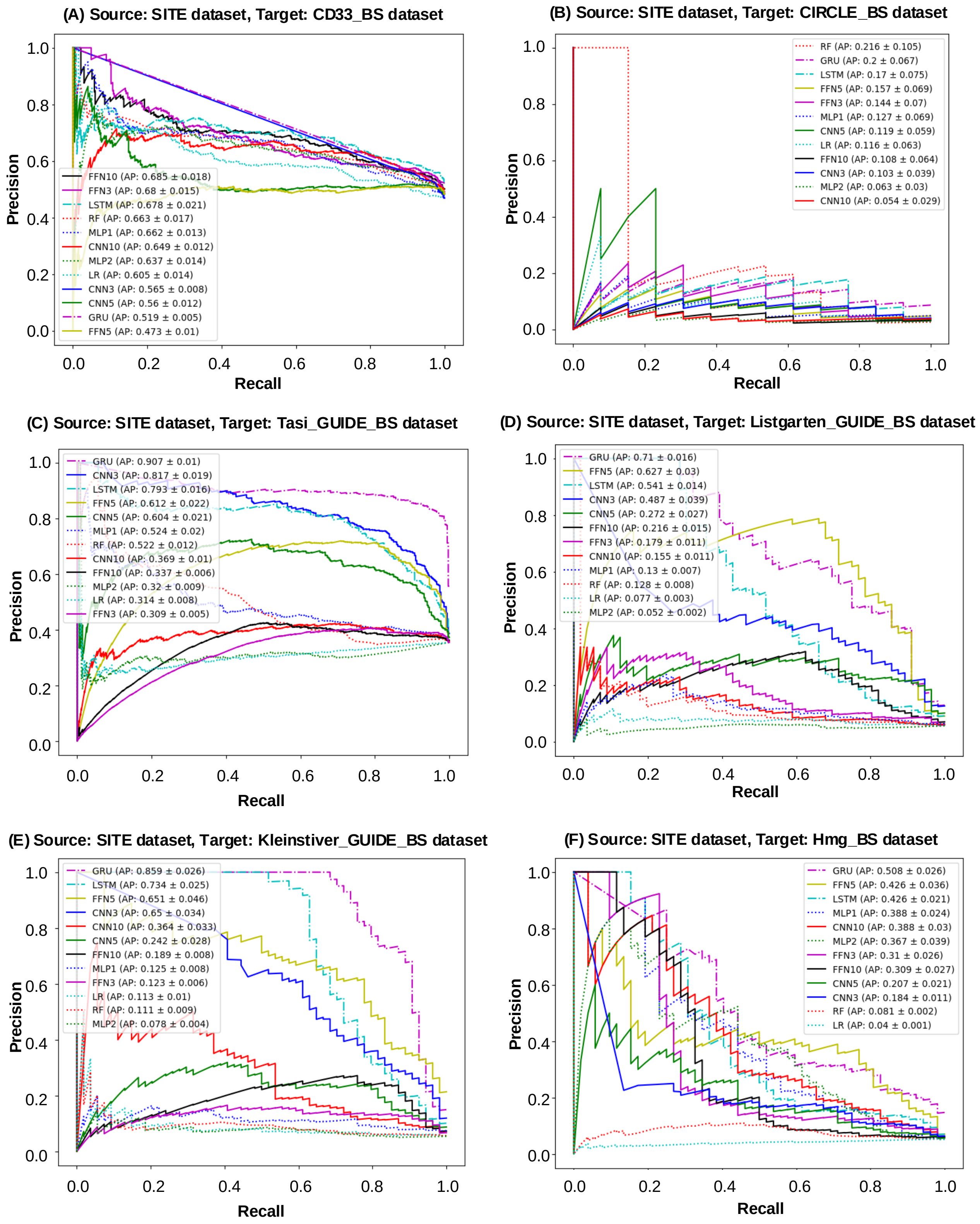

Figure D: Precision-Recall curves for model evaluation - SITE dataset. Precision-Recall curves for the SITE dataset, used as source, and six bootstrapped datasets: CD33_BS, CIRCLE_BS, Tasi_GUIDE_BS, Listgarten_GUIDE_BS , Kleinstiver_GUIDE_BS, Hmg_BS, used as target. The Precision values for each model are displayed in descending order within each figure.
