# Supplementary material for: Similarity-based transfer learning with deep learning networks for accurate CRISPR-Cas9 off-target prediction
Source: PLoS Comput Biol. 2025 Oct 24;21(10):e1013606. doi: 10.1371/journal.pcbi.1013606 (PMC12571277; doi:10.1371/journal.pcbi.1013606)
Supplement: Attachment — Submitted filename: PLOS_CB_rebuttal.pdf [file pcbi.1013606.s004.pdf]

Dear Drs. Lun Hu and Ilya Ioshikhes, dear Reviewers,

We sincerely appreciate your constructive feedback on our manuscript (PCOMPBIOL-D-25-01030) entitled "Similarity-based transfer learning with deep learning networks for accurate CRISPR-Cas9 off-target prediction", authored by Jeremy Charlier, Zeinab Sherkatghanad, and Vladimir Makarenkov. We have carefully addressed each comment made by Reviewers 1 and 2 and incorporated the suggested revisions into the revised manuscript. In this rebuttal letter, we provide detailed responses to these comments and outline the corresponding changes made in the manuscript. To facilitate the review, all revisions in the updated manuscript have been highlighted in red.

Please note that the comments of Reviewer 3 don't seem to correspond to the content of our manuscript – please see below our communication with the journal assistant Olena Szabo, who suggested the following:

“Since the reviewer did not response to our request, please just ignore the comments of this reviewer. The comments from the other two reviewers are sufficient for you to revise your manuscript accordingly. ”

Sincerely yours,

Vladimir Makarenkov (on behalf of all authors)
Professor (full)
Director of DESS graduate program in Bioinformatics
Department of Computer Science
University of Quebec in Montreal
Box 8888, succursale Centre-ville, Montreal (Quebec) Canada - H3C 3P8
and
Mila - Quebec AI Institute, Montreal, QC, Canada
Tel: 1 (514) 987-3000, ext. 3870
Email: makarenkov.vladimir@uqam.ca

PCOMPBIOL-D-25-01030
Similarity-based transfer learning with deep learning networks for accurate CRISPR-Cas9 off-target prediction
PLOS Computational Biology

Dear Dr. Makarenkov,

Thank you for submitting your manuscript to PLOS Computational Biology. After careful consideration, we feel that it has merit but does not fully meet PLOS Computational Biology's publication criteria as it currently stands. Therefore, we invite you to submit a revised version of the manuscript that addresses the points raised during the review

process.

Please submit your revised manuscript within 60 days Aug 25 2025 11:59PM. If you will need more time than this to complete your revisions, please reply to this message or contact the journal office at ploscompbiol@plos.org. When you're ready to submit your revision, log on to https://www.editorialmanager.com/pcompbiol/ and select the 'Submissions Needing Revision' folder to locate your manuscript file.

Please include the following items when submitting your revised manuscript:

* A rebuttal letter that responds to each point raised by the editor and reviewer(s). You should upload this letter as a separate file labeled 'Response to Reviewers'. This file does not need to include responses to formatting updates and technical items listed in the 'Journal Requirements' section below.
* A marked-up copy of your manuscript that highlights changes made to the original version. You should upload this as a separate file labeled 'Revised Manuscript with Track Changes'.
* An unmarked version of your revised paper without tracked changes. You should upload this as a separate file labeled 'Manuscript'.

If you would like to make changes to your financial disclosure, competing interests statement, or data availability statement, please make these updates within the submission form at the time of resubmission. Guidelines for resubmitting your figure files are available below the reviewer comments at the end of this letter

We look forward to receiving your revised manuscript.

Kind regards,

Lun Hu
Academic Editor
PLOS Computational Biology

Ilya Ioshikhes
Section Editor
PLOS Computational Biology

Reviewers' comments:

Reviewer's Responses to Questions

Comments to the Authors:
Please note here if the review is uploaded as an attachment.

Reviewer #1: This manuscript focuses on showing the impact of transfer learning on CRISPRCas9 off-target identification. The number of used similarity metrics are enough. The used datasets are comprehensive. The problem has been extensively studied before, but transfer learning is the main novelty here. Even though the manuscript is written quite well, I have the following comments:
Thank you for your positive comments.

Major Comments:
1- Why did we use 7 in matrix? It is not clear to me as discussed at the top of page 11.
The following detailed explanation regarding this point was added to Section 1.7: "It is worth noting that the number of rows, i.e. 7, in the matrix corresponds to the number of bits used to encode a given sgRNA-DNA sequence pair, including a five-bit character channel (i.e. A, G, C, T, and _) and a two-bit direction channel (to indicate if they appear in the sgRNA or DNA sequence). The five-bit channel encodes the presence-absence of the four nucleotides and insertions/deletions, whereas the direction channel identifies the location of the mismatched nucleotides or insertions/deletions, if any (see Fig. 2 for three examples of such a sequence encoding). We should highlight that 7 is the minimum number of rows one can use to encode the presence-absence of the A, G, C, T, and _ characters in two sequences, including their location information, without any information loss."

2- Can you explain in a bit more detail why MLP surprisingly performs better than sequence-based models such as GRU?
The following new paragraph has been added to the Conclusion section of the revised manuscript to address this comment: "The fact that in many instances two simple MLP models outperformed much more sophisticated RNN-GRU and, especially, RNN-LSTM neural network architectures is not very surprising since MLPs usually cope well with tabular data, such as our CRISPR-Cas9 one-hot encoded sequence datasets, allowing for capturing complex, non-linear patterns, whereas RNN-based models excel at capturing time-series patterns and long-term dependencies in complex scenarios, being particularly useful in natural language processing and speech recognition."

Minor Comments:
1- Can you mathematically describe the evaluation metrics?

All evaluation metrics used have been mathematically described in the new Section 2.2 added to the revised manuscript.

2- The number of provided references are enough. But, in terms of transfer learning and transformers on financial domain, can you cite the following paper as well?
a- Gezici, A.H.B. and Sefer, E., 2024. Deep transformer-based asset price and direction prediction. IEEE Access, 12, pp.24164-24178

The following paragraph and citations, including the suggested one, have been added to the Conclusion section of the revised manuscript: "In the future, we plan to compare our approach with transformer-based models optimized for tabular data \cite{gezici2024deep} as well as with different data augmentation techniques allowing for better leverage of limited datasets \cite{mumuni2022data}."

Reviewer #2: 1. Strengthen Introduction Regarding Algorithmic Novelty:
While you effectively highlight the challenge of data limitations and the benefit of transfer learning, more explicitly state that existing transfer learning applications in CRISPR-Cas9 (e.g., DeepCRISTL, C-RNNCrispr) often lack a principled method for source dataset selection. This will better position your similarity-based pre-evaluation as the key innovation, rather than claiming novelty in the transfer learning algorithms themselves. Clarify that your contribution is in optimizing the transfer learning process through intelligent source selection, not in inventing new deep learning architectures for transfer.

Thank you for your very constructive comments. Following your recommendations, we added new explanations highlighting our main contribution to the abstract, Introduction, and Conclusion sections of the revised manuscript (please see the new text in red in the abstract, the end of Introduction, and the beginning of Conclusion).

2. Elaborate on Data Representation and Distance Metrics (Methods):
Theoretical Basis for Distance Metrics: In Section 1.7, beyond presenting the formulas, delve deeper into the theoretical reasons why cosine, Euclidean, and Manhattan distances might behave differently with your specific 7×L encoded sgRNA-DNA sequence pairs, which are flattened into 7L vectors. Discuss how each metric responds to features like point-wise mismatches, insertions, or deletions given your encoding strategy (e.g., cosine's sensitivity to direction in high-dimensional sparse spaces, Euclidean's sensitivity to magnitude, Manhattan's robustness to outliers). This will provide a more sophisticated understanding of why cosine distance ultimately performs best.

Thank you for this comment. To respond to it, the following new paragraph was added to Section 1.7 of the revised manuscript:

"It is important to note that cosine, Euclidean, and Manhattan distances compared in our study have different strengths and disadvantages. For example, cosine distance is sensitive to direction in high-dimensional sparse spaces. It is used when the magnitude of the vectors is not important. This is the case of binary data such as our sgRNA-DNA sequence pairs encoded as 7L binary vectors. With cosine distance the difference in values is not fully taken into account, but this is not a disadvantage in our settings since this difference can be either 0 or 1 with our sequence encoding. Theoretically, cosine distance should work better

in cases when the encoded sgRNA-DNA sequence pairs have more matching nucleotides, than in cases with frequent insertions, deletions, and mismatches, since better matching sequences would lead to more sparse binary spaces. Euclidean distance is certainly the most natural distance choice as it is directly computed from the cartesian coordinates of the points using the Pythagorean theorem. Euclidean distance is sensitive to magnitude but, as specified above, this is not of importance in our binary settings. This distance usually works well with low-dimensional data. Manhattan distance is known for its robustness to outliers. It is less intuitive than Euclidean distance but works well with discrete and binary components as it considers the veritable path that can be taken within values of those components. Thus, in our settings, we could expect that Manhattan distance treats equally well binary encoded matching nucleotides, insertions, deletions, and mismatches.”.

Normalization Details: Clearly specify the exact Min-Max normalization formula used for "1-normalized_average_distance" in Section 1.7 or a new "Data Processing" subsection. Explicitly state whether this normalization is applied per distance metric independently or across all metrics collectively. This is crucial for reproducibility and transparent interpretation of "similarity values" in Table 3.

The detailed procedure and the exact formulas used are now specified in Section 2.1 – please see the new paragraph in red starting with the sentence “Each similarity estimate appearing in Table 3 was computed …”.

3. Detailed Justification of Sampling and Bootstrapping Strategies (Methods):
Provide a clearer rationale for choosing a bootstrapped target dataset size of 250. Is this based on empirical observation of minimum samples needed for stable predictions, or a specific computational constraint? Similarly, justify the choice of n=5,000 iterations for random sampling within source datasets for distance calculation. Explain how these choices balance computational feasibility with the statistical representativeness of the similarity assessment, particularly in relation to the original large datasets.

The following paragraph was added to the revised manuscript to address this important point:

“The size of each bootstrapped target dataset was 250, while the number of iterations (i.e. comparisons of each target element with the source elements used to assess the distance between the source and target datasets) was set to 5,000. As we determined experimentally, with this number of iterations (denoted by n_itr in Algorithm 1 below), the average distance between the source and target datasets found by the random search converges towards the distance provided by an exhaustive search algorithm. In the large majority of cases, this number of iterations was sufficient to achieve two-digit precision after the decimal point during the distance calculation. This allowed us to obtain reliable results without having to run the computations for several days.

Regarding the size of 250 of the bootstrapped targets, it was selected to see how the proposed methodology would work with some high-quality CRISPR-Cas9 datasets of realistically small size. For example, in a recent Nature Communication paper, Ham et al. {ham2023generalizable} used the TevSpCas9 dataset with 279 samples as well as the SpCas9 dataset with 303 samples to conduct their transfer learning experiments with a novel machine learning architecture (crisprHAL) meant to improve sgRNA activity prediction."

4. In-depth Discussion of Similarity-Performance Link (Results and Discussion): Interpreting Metric Discrepancies: While you note that Euclidean and Manhattan distances provide larger differences between recommended and non-recommended sources but cosine gives higher overall similarity, delve deeper into why this occurs. For instance, do Euclidean/Manhattan distances, being sensitive to absolute differences, better highlight stark dissimilarity, even if overall magnitude is less important for transferability than feature direction (captured by cosine)? Connect these observations more directly to the underlying biological or sequence characteristics.

The following new paragraph (see Section 2.1) was added to the revised manuscript to respond to this important question: "This means that Euclidean and Manhattan distances, being sensitive to absolute differences, better highlight stark dissimilarity between datasets. However, our findings suggest that overall magnitude is less important for transferability than feature direction, which is captured by cosine distance (see Section 2.3). Such a result should be related to the binary nature of the encoded sgRNA-DNA sequence pairs since in our settings even the vectors with different locations of matches, mismatches, and indels are sparse enough to have at least 50% of matching 0 values, thus leading to the lowest cosine similarity values that are slightly higher than 0.5. In the case of Euclidean and Manhattan distances, the lowest normalized distance values can be close to 1, leading to the corresponding similarity values that are slightly higher than 0. Obviously, the maximum similarity value is limited by 1 (in case of a perfect sequence match) for all three metrics considered.".

Illustrating Negative Transfer Avoidance: Explicitly discuss how your similarity-based pre-selection strategy mitigates negative transfer. Can you point to instances where a source dataset, if chosen solely by size or availability (without similarity assessment), would have led to suboptimal or negative transfer, and how your method successfully avoided this? Providing even a hypothetical or qualitative example could strengthen this point.

Thank you for this suggestion. An example of a negative transfer was added to the revised manuscript (see Section 2.3):

"Moreover, our results clearly demonstrate that similarity-based source data pre-selection is necessary to mitigate negative knowledge transfers. If a source dataset is chosen solely by size, availability, or even class imbalance ratio, but without similarity assessment, this could eventually lead to a suboptimal or negative transfer. For example, the CIRCLE and SITE datasets considered in our study have comparable sizes and almost identical class

imbalance ratios (0.0128 and 0.0176, respectively - see Table 1), but a knowledge transfer from CIRCLE to SITE as well as that from SITE to CIRCLE are clearly negative with the highest F1-score values of 0.1345 (see Table 5) and 0.1008 (see Table 6), respectively, over all competing ML and DL models. Our similarity-based analysis suggests that such transfers should be avoided (see Table 3)."

Performance Beyond Similarity: While you effectively demonstrate that high cosine similarity often correlates with top model performance, also discuss cases where a source dataset with slightly lower cosine similarity might still yield competitive or even superior performance for specific models due to other factors (e.g., a richer representation of specific rare patterns important for the target task, or the specific model's inductive biases aligning better with that particular source's data distribution). This would add nuance to the "reliability" claim.

The suggested cases were discussed in the following new paragraph added to Section 2.3: "It is worth noting that in some cases a potential source dataset with a slightly lower cosine similarity with the target might still yield competitive or even superior performance for specific models - MLP-based models in our case. This could be due to such factors as a richer representation of specific rare patterns important for the target task or some MLP inductive biases aligning better with the source data distribution. For example, the recommended transfers with lower cosine similarity scores of 0.5701 from CIRCLE to Listgarten GUIDE BS and of 0.5672 from CIRCLE to Kleinstiver GUIDE BS led to competitive knowledge transfers with the corresponding best F1-score score values of 0.5646 and 0.7048, obtained, respectively, with MLP1 and MLP2 (see Table 5). It was not so for Euclidean and Manhattan similarities whose highest values in this case led to transfers from CD33 (instead of CIRCLE) with much lower best F1-score score values of 0.2500 for Listgarten GUIDE BS and of 0.1946 for Kleinstiver GUIDE BS, both obtained with MLP1 (see Table 4)".

5. Refine Conclusion and Future Work:
Concrete Future Directions: Beyond proposing "more advanced similarity measures" or "adaptive transfer learning strategies", offer concrete examples. For instance, could your similarity framework be extended to incorporate biological contextual information (e.g., cell type, experimental conditions) beyond just sequence similarity? Could new deep learning architectures for domain adaptation (which explicitly address distribution shifts between source and target) be integrated with your similarity-based selection?
Broader Impact: Elaborate on the broader impact of your dual-layered framework. How specifically does it "streamline the transfer learning process"? Does it reduce trial-and-error, save computational resources, or enable faster development of CRISPR-Cas9 off-target prediction models for new experimental data?

Thank you for this comment. To address it, we added the three following new paragraphs to the Conclusion section of the revised manuscript:

"Broader impacts of the proposed dual-layered framework are the following: (1) The new framework streamlines the transfer learning process by reducing the number of potential

source datasets and recommended ML and DL models, and thus the number of trial-and-error attempts, which are convenient for a selected target dataset, and (2) it enables faster development of transfer learning models for CRISPR-Cas9 off-target prediction, which can now be successfully tested on mutually compatible sets (i.e. those with high cosine similarity scores) of source and target data.

Moreover, it would be interesting to extend the proposed similarity framework beyond sequence similarity by incorporating into it available biological and experimental information, such as species, cell type, enzyme type, experimental conditions/technology being used, and data size. Specifically, each of these factors could be added to the discussed 7L input vectors as an extra component, normalized to [0,1] range for numerical data and one-hot encoded for categorical data.

It would also be interesting to integrate the proposed similarity-based selection with deep learning architectures for domain adaptation that explicitly address distribution shifts between source and target ({SUN201584, rozantsev2018beyond}). In this case, the source labeled sample weights could ideally be calculated leveraging both distribution and similarity patterns of the source and target samples."

6. Minor Revisions:
Figure Titles and Labels: Ensure consistency between figure titles and the text. For example, Figure 4's subplot titles refer to "distance" (e.g., "Cosine distance for CD33 dataset") , while the main text and Table 3 refer to "similarity" (which is 1 - normalized distance). Please unify this terminology to avoid confusion. Suggestion: "Cosine Similarity (1 - Normalized Distance) for CD33 dataset".

The suggested correction was applied to the captions of Table 3 and Figure 4.

Reviewer #3: The work has written well and has significance merits but there are minor changes are required for acceptance.

1. Clarify what the term "discriminator score" refers to in the dual anomaly scoring mentioned in the abstract.
2. Rewrite the opening sentence of the introduction for brevity and improved clarity.
3. Reformat the literature review section into bullets or a table to clearly highlight key study comparisons.
4. Ensure consistent usage of the term "self-adaptive" throughout the manuscript.
5. Revise lengthy sentences for better grammar and readability, particularly in the introduction.
6. Add a summary paragraph highlighting similarities and differences among reviewed studies to contextualize SAD-GAN's contribution.
7. Define and explain all mathematical symbols (α, β, G, D) at their first mention for clarity.
8. Elaborate on how alerts are triggered and communicated during anomaly detection.
9. Justify the use of non-overlapping time windows in Section 3.4 by comparing with

alternatives like sliding windows.
10. Provide details on how static and dynamic thresholds are implemented in Section 4.5.
11. Clarify how the system enables autonomous anomaly detection and response without prior planning.
12. Include a discussion on the limitations or drawbacks of the proposed approach.
13. Expand the future work section by outlining 4–5 specific research directions.

This seems to be a review submitted by mistake, so we ignored it. Please see below our communication with the journal assistant Olena Szabo.

Case Number: 09086423
Dear Dr Makarenkov,

I am getting back to you after having heard back from the editor.

Since the reviewer did not response to our request, please just ignore the comments of this reviewer. The comments from the other two reviewers are sufficient for you to revise your manuscript accordingly.

We are looking forward to your revisions.

Kind regards,

Olena Szabo
Journal Assistant, Editorial Office Ltd.
On behalf of PLOS Computational Biology

Dear Dr Makarenkov,

Thank you for your query, apologies for these inconveniences.
I have contacted the editor, and they confirmed that is a wrong review.

I have contacted the reviewer and asked them to send us the correct review.

I will get back to you as soon as we have any developments here.

Kind regards,

Olena Szabo
Journal Assistant, Editorial Office Ltd.
On behalf of PLOS Computational Biology

Case Number: 09086423

--------------- Original Message ---------------
From: Vladimir Makarenkov [makarenkov.vladimir@uqam.ca]
Sent: 30/06/2025, 19:08
To: ploscompbiol@plos.org; hulun@ms.xjb.ac.cn
Subject: Comments of Reviewer 3 (Dr. Surjeet Dalal) - Re: Decision from PLOS Computational Biology (PCOMPBIOL-D-25-01030) - [EMID:1b158ccce63b441a]

Dear Dr. Lun Hu,

Many thanks for accepting to handle the revision of our manuscript submitted to PLOS Computational Biology:
MS: PCOMPBIOL-D-25-01030
"Similarity-based transfer learning with deep learning networks for accurate CRISPR-Cas9 off-target prediction"

We attentively studied the comments of Reviewer 3 (Dr. Surjeet Dalal - please see them below) and it seems that they don't completely correspond to the content of our manuscript.

For example:
We don't have the expression "discriminator score" in the abstract, don't use sliding windows in our algorithm, and don't have Sections 3.4 and 4.5 in our manuscript.

Maybe, by mistake, Dr. Surjeet Dalal sent to the journal a review made for another paper ?

Could you please contact him to see if it was the case ?

Thank you,

Vladimir Makarenkov

Have the authors made all data and (if applicable) computational code underlying the findings in their manuscript fully available?
The PLOS Data policy requires authors to make all data and code underlying the findings described in their manuscript fully available without restriction, with rare exception (please refer to the Data Availability Statement in the manuscript PDF file). The data and code should be provided as part of the manuscript or its supporting information, or deposited to a public repository. For example, in addition to summary statistics, the data points behind means, medians and variance measures should be available. If there are restrictions on publicly sharing data or code —e.g. participant privacy or use of data from a third party— those must be specified.

Reviewer #1: Yes

Reviewer #2: None

Reviewer #3: Yes

PLOS authors have the option to publish the peer review history of their article (what does this mean?). If published, this will include your full peer review and any attached files.
If you choose "no", your identity will remain anonymous but your review may still be made public.
Do you want your identity to be public for this peer review? For information about this choice, including consent withdrawal, please see our Privacy Policy.

Reviewer #1: **Yes:** Emre Sefer

Reviewer #2: No

Reviewer #3: **Yes:** surjeet dalal

Figure resubmission:

While revising your submission, please upload your figure files to the Preflight Analysis and Conversion Engine (PACE) digital diagnostic tool, https://pacev2.apexcovantage.com/. PACE helps ensure that figures meet PLOS requirements. To use PACE, you must first register as a user. Registration is free. Then, login and navigate to the UPLOAD tab, where you will find detailed instructions on how to use the tool. If you encounter any issues or have any questions when using PACE, please email PLOS at figures@plos.org. Please note that Supporting Information files do not need this step. If there are other versions of figure files still present in your submission file inventory at resubmission, please replace them with the PACE-processed versions.

Reproducibility:
To enhance the reproducibility of your results, we recommend that authors of applicable studies deposit laboratory protocols in protocols.io, where a protocol can be assigned its own identifier (DOI) such that it can be cited independently in the future.
Additionally, PLOS ONE offers an option to publish peer-reviewed clinical study protocols. Read more information on sharing protocols at https://plos.org/protocols?utm_medium=editorial-email&utm_source=authorletters&utm_campaign=protocols

---

In compliance with data protection regulations, you may request that we remove your personal registration details at any time. (Remove my information/details). Please contact the publication office if you have any questions.
